# Supplementary material for: Hybrid Models and Biological Model Reduction with PyDSTool
Source: PLoS Comput Biol. 2012 Aug 9;8(8):e1002628. doi: 10.1371/journal.pcbi.1002628 (PMC3415397; doi:10.1371/journal.pcbi.1002628)
Supplement: Text S4 — Complete source code for the PyDSTool package (version 0.88.120504). Includes API documentation and help files linking to web pages. This file is identical to the current public release on Sourceforge.net. (ZIP) [file pcbi.1002628.s004.zip › PyDSTool/html/PyDSTool.Interval'.IntervalMembership-class.html]

xml version="1.0" encoding="ascii"?


PyDSTool.Interval'.IntervalMembership


| Home | Trees | Indices | Help | PyDSTool |
| --- | --- | --- | --- | --- |

Package PyDSTool ::
Module Interval' ::
Class IntervalMembership

[hide private]

[frames] | no frames]

# Class IntervalMembership

source code

```
object --+    
         |    
       int --+
             |
            IntervalMembership
```

---

Numeric Interval membership type.


|  |  |
| --- | --- |
| Instance Methods | [hide private] |

|  |  |
| --- | --- |
| \_\_and\_\_(self, v)  Interval `logical AND` for checking interval containment. | source code |

|  |  |
| --- | --- |
| \_\_init\_\_(self, \*arg)  x.\_\_init\_\_(...) initializes x; see x.\_\_class\_\_.\_\_doc\_\_ for signature | source code |

|  |  |
| --- | --- |
| \_\_or\_\_(self, v)  Interval `logical OR` for checking interval intersection. | source code |

|  |  |
| --- | --- |
| \_\_rand\_\_(self, v)  y&x | source code |

|  |  |
| --- | --- |
| \_\_repr\_\_(self)  str(x) | source code |

|  |  |
| --- | --- |
| \_\_ror\_\_(self, v)  y\|x | source code |

|  |  |
| --- | --- |
| \_\_str\_\_(self)  str(x) | source code |

**Inherited from `int`**:
`__abs__`,
`__add__`,
`__cmp__`,
`__coerce__`,
`__div__`,
`__divmod__`,
`__float__`,
`__floordiv__`,
`__getattribute__`,
`__getnewargs__`,
`__hash__`,
`__hex__`,
`__index__`,
`__int__`,
`__invert__`,
`__long__`,
`__lshift__`,
`__mod__`,
`__mul__`,
`__neg__`,
`__new__`,
`__nonzero__`,
`__oct__`,
`__pos__`,
`__pow__`,
`__radd__`,
`__rdiv__`,
`__rdivmod__`,
`__rfloordiv__`,
`__rlshift__`,
`__rmod__`,
`__rmul__`,
`__rpow__`,
`__rrshift__`,
`__rshift__`,
`__rsub__`,
`__rtruediv__`,
`__rxor__`,
`__sub__`,
`__truediv__`,
`__xor__`

**Inherited from `object`**:
`__delattr__`,
`__reduce__`,
`__reduce_ex__`,
`__setattr__`


|  |  |
| --- | --- |
| Properties | [hide private] |

**Inherited from `object`**:
`__class__`


|  |  |
| --- | --- |
| Method Details | [hide private] |

|  |  |
| --- | --- |
| \_\_and\_\_(self, v)  *(And operator)* | source code |

Interval `logical AND` for checking interval containment. e.g. if both
endpoints are contained then the whole interval is.

Overrides: int.\_\_and\_\_

|  |  |
| --- | --- |
| \_\_init\_\_(self, \*arg)  *(Constructor)* | source code |

x.\_\_init\_\_(...) initializes x; see x.\_\_class\_\_.\_\_doc\_\_ for
signature

Overrides: object.\_\_init\_\_: *(inherited documentation)*

|  |  |
| --- | --- |
| \_\_or\_\_(self, v)  *(Or operator)* | source code |

Interval `logical OR` for checking interval intersection. e.g. if at
least one endpoint is contained then there is intersection.

Overrides: int.\_\_or\_\_

|  |  |
| --- | --- |
| \_\_rand\_\_(self, v) | source code |

y&x

Overrides: int.\_\_rand\_\_: *(inherited documentation)*

|  |  |
| --- | --- |
| \_\_repr\_\_(self)  *(Representation operator)* | source code |

str(x)

Overrides: object.\_\_repr\_\_: *(inherited documentation)*

|  |  |
| --- | --- |
| \_\_ror\_\_(self, v) | source code |

y|x

Overrides: int.\_\_ror\_\_: *(inherited documentation)*

|  |  |
| --- | --- |
| \_\_str\_\_(self)  *(Informal representation operator)* | source code |

str(x)

Overrides: object.\_\_str\_\_: *(inherited documentation)*

  


| Home | Trees | Indices | Help | PyDSTool |
| --- | --- | --- | --- | --- |

|  |  |
| --- | --- |
| Generated by Epydoc 3.0.1 on Fri May 4 15:24:06 2012 | http://epydoc.sourceforge.net |
